# Identification of EGF-NF-κB-FOXC1 signaling axis in basal-like breast cancer

**DOI:** 10.1186/s12964-017-0180-3

**Published:** 2017-06-19

**Authors:** Stacey Chung, Yanli Jin, Bingchen Han, Ying Qu, Bowen Gao, Armando E. Giuliano, Xiaojiang Cui

**Affiliations:** 1 Department of Surgery, Samuel Oschin Cancer Institute, Cedars-Sinai Medical Center, California, Los Angeles 90048 USA; 2 Cedars-Sinai Medical Center, Davis Research Building 2065, 8700 Beverly Blvd, California, Los Angeles 90048 USA

**Keywords:** FOXC1, Epidermal growth factor, NF-κB, Basal-like breast cancer

## Abstract

**Background:**

The pathogenesis of human basal-like breast cancer (BLBC) is not well understood and patients with BLBC have a poor prognosis. Expression of the epidermal growth factor receptor (EGFR) and nuclear factor-κB (NF-κB) is well-known to be upregulated in BLBC. The forkhead box C1 (FOXC1) transcription factor, an important prognostic biomarker specific for BLBC, has been shown to be induced by EGF and is critical for EGF effects in breast cancer cells. How FOXC1 is transcriptionally activated in BLBC is not clear.

**Methods:**

Luciferase reporter assays were performed to show that NF-κB-p65 enhances FOXC1 promoter activity in BLBC cells (MDA-MB-468). Electrophoretic mobility shift assay, biotinylated oligonucleotide precipitation assay, and chromatin immunoprecipitation assay were used to show that NF-κB interacts and binds to the promoter region of FOXC1.

**Results:**

In this study, we demonstrate that NF-κB is a pivotal mediator of the EGF/EGFR regulation of FOXC1 expression by binding to the FOXC1 promoter to activate FOXC1 transcription. Loss or inhibition of NF-κB diminished FOXC1 expression.

**Conclusion:**

Collectively, our findings reveal a novel EGFR-NF-κB-FOXC1 signaling axis that is critical for BLBC cell function, supporting the notion that intervention in the FOXC1 pathway may provide potential modalities for BLBC treatment.

**Electronic supplementary material:**

The online version of this article (doi:10.1186/s12964-017-0180-3) contains supplementary material, which is available to authorized users.

## Background

Breast cancer is grouped into four major molecular subtypes that include luminal A, luminal B, human epidermal growth factor receptor 2-enriched (HER2+) and basal-like breast cancer (BLBC) [1]. BLBC was shown to have low expression of the estrogen receptor (ER), progesterone receptor (PR) and HER2 gene, which encompasses 15–20% of all invasive breast cancers [2]. Patients diagnosed with BLBC present with aggressive clinical features, such as metastasis to the lung and brain, high histologic grade and have a poor prognosis [3, 4]. Currently, the only form of treatment for BLBC is chemotherapy.

The forkhead box C1 (FOXC1) transcription factor was initially shown to be important for development of the brain, heart and eye during embryonic development [5]. However, it is also overexpressed in many different types of cancer including breast, hepatocellular carcinoma, prostate, pancreatic, and non-small cell lung cancer [6–11]. It was previously shown that activation of the epidermal growth factor receptor (EGFR), an established BLBC marker, upregulates FOXC1 expression in BLBC cells through Ras/ERK and PI3K/AKT-mediated pathways [12]. Inhibition of FOXC1 expression reduces cell migration and invasion that is induced by EGF, while inhibition of EGFR lowers FOXC1 expression and abrogates tumor growth in mice [12]. Moreover, EGF-induced FOXC1 expression occurs not only in breast cancer cells but also in prostate cancer cells [7, 12].

The mechanism of how FOXC1 is regulated in BLBC is still unclear. We want to understand what processes are upstream of FOXC1 in the hope of elucidating the biological basis of BLBC development. Toward this goal, we seek to identify the regulators that link between microenvironmental cues, such as EGF, and FOXC1 expression. In this study, we demonstrate that NF-κB transcriptionally regulates FOXC1 through an EGF-mediated signaling pathway by binding to the promoter region of FOXC1. Our results suggest that FOXC1 may serve as a readout of EGF-NF-κB signaling activity in breast cancer.

## Results

### NF-κB transcription factor is essential for the EGF induction of FOXC1

**Fig. 1:**
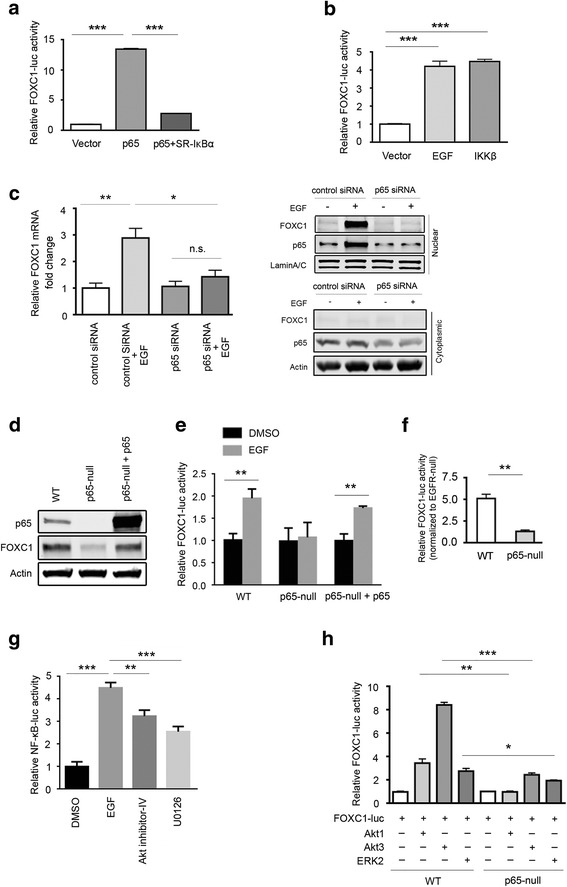
NF-κB transcription factor mediates EGF-induced FOXC1 expression. **a** MDA-MB-468 cells were transiently co-transfected with the FOXC1 promoter-luc and NF-κB (p65), IκBα S32A/S36A super-repressor (p65 + SR-IκBα), or the vector. Reporter activities were assessed by luciferase assays. ***, *P* < 0.0001. **b** MDA-MB-468 cells were transiently transfected with the FOXC1 promoter-luc and IKKβ construct or treated with 100 ng/mL EGF for 24 h, followed by luciferase assays. ***, *P* < 0.0001. Data represent mean ± SD from 3 independent experiments. **c** Left, MDA-MB-468 cells were transfected with p65 siRNA for 48 h and then treated with or without EGF for 6 h. FOXC1 mRNA levels were examined using qRT-PCR. n.s., not significant; **, *P* < 0.001; *, *P* < 0.05. Right, MDA-MB-468 cells were transfected with p65 siRNA for 48 h and then treated with or without EGF for another 24 h. FOXC1 and p65 in nuclear and cytoplasmic fractions were examined using immunoblotting. Lamin A/C was used as a nuclear marker and actin was used as a cytoplasmic marker. **d** WT, p65-null, and p65-reconstituted MEFs were transfected with the FOXC1 promoter-luc and immunoblotted for p65 protein expression and then **(e)** *,* treated with EGF or vehicle for 24 h, followed by luciferase assays. **, *P* < 0.001; ***, *P* < 0.0001. **f** WT or p65−/− MEFs were co-transfected with the FOXC1 promoter-luc and pBABE-EGFR construct, followed by luciferase assays. **, *P* < 0.001. Data represent mean ± SD of 3 independent experiments. **g** MDA-MB-468 cells were transiently transfected with the NF-κB-luc construct. After 30 min pre-treatment with 5 μM U0126 (MEK inhibitor) or 1 μM AKTIV (AKT inhibitor), cells were stimulated with EGF for 24 h in the presence or absence of the inhibitors. Reporter activities were measured by luciferase assays. **, *p* < 0.01; ***, *P* < 0.0001. **h** Wild-type and p65−/− MEFs were co-transfected with the FOXC1 promoter reporter construct and constitutively active Akt1, Akt3 or ERK2 constructs for 24 h. FOXC1 promoter activity was assessed by luciferase assays. *, *P* < 0.05; **, *P* < 0.01; ***, *p* < 0.0001

We previously found that EGF regulates FOXC1 expression through Ras/ERK and PI3K/Akt-mediated pathways in BLBC cells [12]. However, it is not known which transcription factor mediates this event. Nuclear factor-κB (NF-κB) is a well-known transcription factor that associates with ER-negative breast cancers, exists mainly in human BLBC cells, and has the highest activity in triple-negative tumors [13–17]. Therefore, NF-κB may play a role in regulating FOXC1. As illustrated in Fig. 1a, overexpression of p65, a subunit of the NF-κB transcription complex, in MDA-MB-468 cells markedly elevated FOXC1 promoter activity, which was abrogated by the IκBα S32A/S36A super-repressor (SR-IκBα). This same trend was also found in MDA-MB-231 and BT-20 cells (Additional file 1: Figure S1A). We also treated MDA-MB-468 cells with EGF to enhance NF-κB activity, resulting in a similar effect on the FOXC1 promoter activity seen when p65 was overexpressed (Fig. 1b). Likewise, transfection of IKKβ, an upstream kinase for p65, activated the FOXC1 promoter (Fig. 1b). As expected, FOXC1 protein levels were increased or decreased by IKKβ or SR-IκBα overexpression, respectively (Additional file 1: Figure S1B). Next, we transfected MDA-MB-468 cells with p65 siRNA to test the effect of EGF-induced FOXC1 expression. Immunoblotting and qRT-PCR indicated that p65 knockdown suppressed the increase of FOXC1 mRNA and protein levels by EGF (Fig. 1c). Similar results were obtained using BT-20 cells, in which pharmacologic inhibition of p65 by Bay 11–7082 significantly reduced the induction of FOXC1 mRNA expression by EGF (Additional file 1: Figure S1C) [12]. This p65-mediated EGF effect was not observed in MDA-MB-231 cells (Additional file 1: Figure S1C) probably due to the low expression level of EGFR in this cell line compared to MDA-MB-468 and BT-20, which have high and moderate levels of EGFR and p-EGFR, respectively (Additional file 1: Figure S1D). These data suggest that NF-κB is required for the induction of FOXC1 by EGFR activation.

In line with these results, we found that FOXC1 protein levels were significantly down-regulated in p65-null mouse embryonic fibroblasts (MEFs) but were restored by reconstituted expression of p65 in these cells (Fig. 1d). EGF induction of FOXC1 promoter activity was diminished in p65-null MEFs, which was also reversed by reconstitution of p65 in p65-null MEFs (Fig. 1e). Moreover, knocking out p65 abolished the activation of the FOXC1 promoter in EGFR-overexpressing MEFs (Fig. 1f). It was previously reported that constitutively active Akt and ERK can induce NF-κB activity [18, 19]. Using a luciferase reporter construct containing the consensus NF-κB site, we found that EGF activated NF-κB-responsive reporter activity, which was attenuated by ERK and Akt inhibitors (Fig. 1g). In agreement with this, the activation of NF-κB by EGF also led to increased phosphorylation of p65 at serine 536 (p-Ser536) in BT20 cells (Additional file 1: Figure S1E). Treating cells with the Akt inhibitor or a combination of the ERK and Akt inhibitors reduced p65 p-Ser536 levels (Additional file 1: Figure S1E). In MEF cells, FOXC1 promoter activity was enhanced by overexpressing Akt1, Akt3, and ERK2 but the induction was significantly reduced in p65-null MEFs (Fig. 1h). Akt is known to directly phosphorylate p65, which may cause the enhanced FOXC1 activity than compared to ERK. In summary, these finding suggest that NF-κB mediates the EGF-induced FOXC1 expression in BLBC cells.

### EGF induces NF-κB binding to the promoter region of FOXC1

**Fig. 2:**
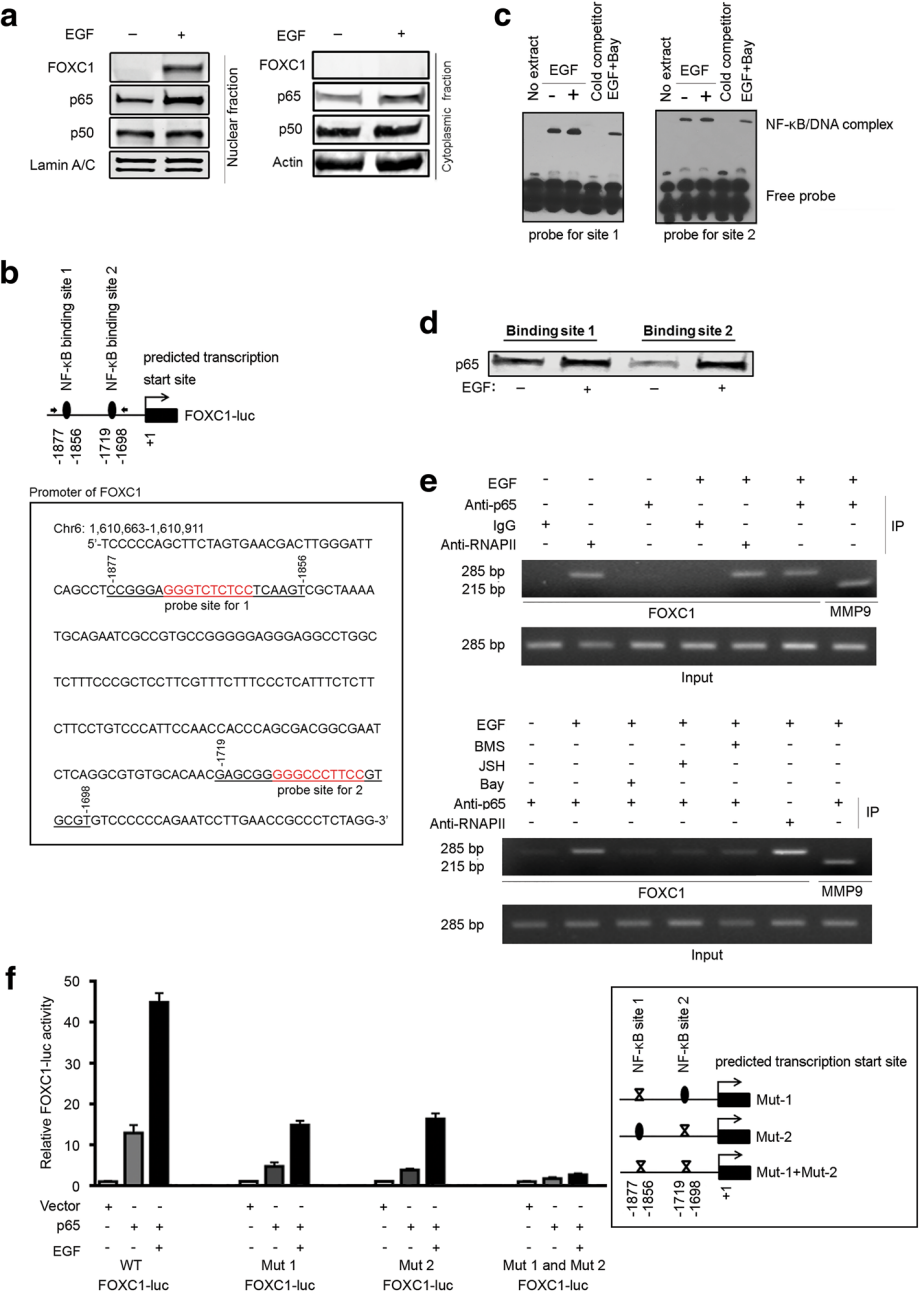
EGF stimulates binding of NF-κB to the promoter region of FOXC1. **a** MDA-MB-468 cells were serum-starved overnight and then treated with EGF for 24 h. FOXC1, p65, p50 levels in nuclear and cytoplasmic fractions were examined using immunoblotting. Lamin A/C was used as a nuclear marker and actin was used as a cytoplasmic marker. **b** Two conserved putative NF-κB binding sites (underlined; −1856 to −1877 and −1698 to −1719) in the cloned FOXC1 promoter. NF-κB probe sites are highlighted in red. **c** MDA-MB-468 cells were serum-starved overnight and treated with EGF for 24 h in the presence or absence of 10 μM Bay 11–7082 (Bay; NF-κB inhibitor) after preincubation with the inhibitor for 1 h. Nuclear protein was extracted. EMSA analysis was conducted using biotin-labeled double-stranded NF-κB probes. **d** MDA-MB-468 cells were serum-starved overnight and treated with EGF for 24 h. Nuclear protein was extracted and mixed with biotin-labeled double stranded NF-κB probes and streptavidin beads. p65-probe interaction was examined with immunoblotting. **e** Top, serum-starved MDA-MB-468 cells were treated with or without EGF for 24 h and fixed by formaldehyde. ChIP assays were performed using p65 antibody to examine the binding of p65 to the FOXC1 promoter. The PCR amplified FOXC1 promoter region is indicated by solid arrow (see the diagram in *B*). Bottom, MDA-MB-468 cells were treated with EGF for 24 h after preincubation with the NF-κB inhibitors for 1 h: 5 μM BMS-345541 (BMS; IKK inhibitor III), 10 μM Bay 11–7082 (Bay; NF-κB Activation Inhibitor II) and 50 μM JSH-23 (JSH; NF-κB Activation Inhibitor II). Then ChIP assays were performed. **f** The insert shows schematic diagrams of the two putative NF-κB binding sites in the FOXC1 promoter in which the two NF-κB binding sites were mutated by site-directed mutagenesis (see Materials and Methods). MDA-MB-468 cells were transfected with the wild-type or mutated FOXC1 promoter and NF-κB constructs. Cells were treated with EGF or vehicle for 24 h, followed by luciferase assays

EGF increased p65 translocation into the nucleus indicated by immunoblotting (Fig. 2a), therefore we assessed whether p65 binds to the FOXC1 promoter in vitro. There are two putative conserved NF-κB binding sites (−1877 and −1719, transcription start site) in the cloned FOXC1 promoter (Fig. 2b). Thus, EMSA was performed using biotin-labeled probes for the two p65 binding sites and nuclear extracts from MDA-MB-468 cells with or without EGF treatment. Unlabeled p65 probes in 200-fold excess were used as cold competitors. As shown in Fig. 2c, EGF stimulation enhanced p65 binding to the probes as indicated by the NF-κB/DNA complex signals. Notably, the cold probes and the p65 inhibitor, Bay 11–7082, partially blocked the EGF-induced p65 binding to the probes. We also performed a biotinylated oligonucleotide precipitation assay by mixing nuclear extracts from MDA-MB-468 cells with or without EGF treatment with biotin-labeled probes of p65 binding sites. Subsequently, these probes were pulled down using streptavidin beads to check for p65 interaction. As shown in Fig. 2d, cells treated with EGF have increased p65 interaction to the probes. To examine whether p65 binds to the FOXC1 promoter in vivo, ChIP assays were performed. As presented in the top panel of Fig. 2e, p65 weakly bound to the FOXC1 promoter, but addition of EGF dramatically increased the recruitment of p65 protein to the FOXC1 promoter. EGF-induced p65 binding to the MMP-9 promoter was used as a positive control [20]. Furthermore, ChIP assays showed that NF-κB pathway inhibitors (BMS-345541, Bay 11–7082, and JSH-23) blocked EGF-elicited p65 binding to the FOXC1 promoter (Fig. 2e, bottom). Consistent with these results, inhibition of p65 by Bay 11–7082 and BMS-345541 also reduced FOXC1 protein expression levels (Additional file 2: Figure S2). Interestingly, RNA polymerase II bound to the FOXC1 promoter in the absence of EGF and this binding was not increased by EGF (Fig. 2e). We postulate that EGF may instead function to recruit or activate essential transcriptional regulators, such as transcription factors (e.g. NF-κB) and coactivators, enabling an active RNA polymerase II transcription complex to start FOXC1 gene transcription.

To determine the essential role of the two p65 binding sites in FOXC1 transcription, we mutated the two p65 binding sites separately or simultaneously in the FOXC1 promoter (Mut 1:CCGGGA*GGG*(CCC)TCTCT*CC*(GG)TCAAGT, Mut 2: GAGCGG*GGG*(CCC)CCCTT*CC*(GG)GTGCGT and Mut 1+ Mut 2) (Fig. 2f, insert). Luciferase assays demonstrated that mutations of site 1 or site 2 decreased the NF-κB-induced FOXC1 promoter activity, while mutations of both site 1 and 2 abolished the effect of NF-κB (Fig. 2f), suggesting that these two binding sites are essential for NF-κB regulation of FOXC1 transcription in BLBC cells.

## Discussion

**Fig. 3:**
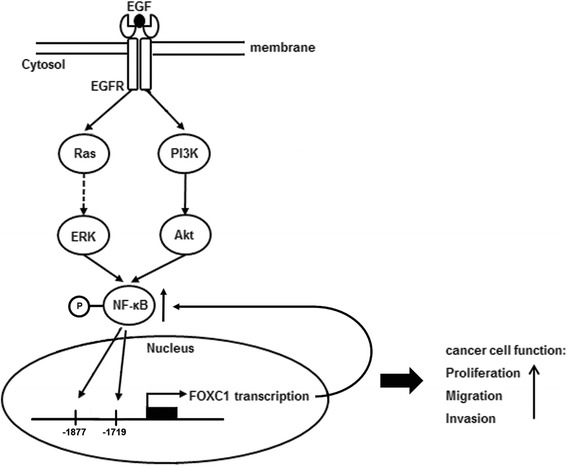
Proposed model of EGF-mediated NFκB-FOXC1 signaling network. The binding of EGF activates the EGFR receptor, leading to downstream activation of Ras-mediated or PI3K-mediated pathways. Activated Ras (Ras-bound GTP) leads to the subsequent phosphorylation and kinase activation sequence of RAF, MEK and ERK, while PI3K phosphorylates PI(4,5)P_2_ to convert it to PI(3,4,5)P_3_, which activates Akt. Activation of ERK and Akt leads to the phosphorylation and activation of NF-κB. Phosphorylated NF-κB enters the nucleus where it binds to the promoter region of FOXC1 to up-regulate transcription and protein expression of FOXC1. In our previous study, we have shown that FOXC1 up-regulates NF-κB activity and expression in BLBC cells [24], which ultimately increases cellular proliferation, migration and invasion

Emerging evidence has established FOXC1 as an important marker and regulator of BLBC development and progression [6, 21]. In this study, we identified that the NF-κB transcription factor regulates FOXC1 expression in BLBC cells through EGFR signaling (Fig. 3). NF-κB is well-established to play a pivotal role in cancer development [22, 23]. Sustained NF-κB activation exists mainly in human BLBC and ER-negative breast cancer as opposed to ER-positive breast cancer [15, 16]. We showed that EGFR activation promotes nuclear translocation of NF-κB, which binds to the FOXC1 promoter elicited by Ras/ERK and PI3K/Akt pathways. This mechanism supports our previous finding that EGFR inhibition reduces active EGFR and FOXC1 levels in xenograft mammary tumors [12]. Thus, relatively low activities of ERK, Akt, and NF-κB may contribute to low FOXC1 levels in non-basal breast tumors. Of note, we have previously shown that FOXC1 regulates NF-κB signaling and that overexpression of FOXC1 increases NF-κB transcription [24]. Inhibition of NF-κB blocks FOXC1-mediated migration, invasion and proliferation [24]. The positive feedback regulatory loop between FOXC1 and NF-κB may explain why both proteins are highly specific to BLBC and implicates an essential role of NF-κB-FOXC1 signaling in BLBC pathogenesis. Other studies described that NF-κB regulates cancer stem cell properties [25], which complements our previous study that FOXC1 also regulates cancer stem cell function through a Hedgehog/Gli-mediated pathway [26]. Reports have shown that cancer stem cell properties are enriched in BLBC compared with other breast cancer subtypes [27]. It is postulated that NF-κB-FOXC1 may be involved in breast cancer stem cell function. Although there are many pathways that regulate BLBC and triple-negative breast cancer, the EGF-NF-κB-FOXC1 signaling axis is presumably specific and essential for BLBC.

It is conceivable that transcription factors other than NF-κB may be involved in the regulation of FOXC1. Luciferase assays using cloned promoter regions have limitations as an in vitro system. The combination of EGF treatment in cells overexpressing p65 showed enhanced FOXC1 promoter activity (Fig. 2e), indicating that other factors in addition to NF-κB may be involved in regulating FOXC1. Therefore, it is important to consider that multiple transcription factors or co-activators may interact with the promoter region of FOXC1 or can directly interact with NF-κB. Moreover, although NF-κB is highly expressed in BLBC cells, it is still expressed in ER-positive and HER2-positive cells whereas FOXC1 is highly specific to BLBC. Thus, other mechanisms are potentially involved in the FOXC1 induction of BLBC. In this study, we focused on using EGF as the main ligand to activate EGFR but other factors including chemokines and cytokines can also activate Akt, ERK, and NF-κB and accordingly may play a role in the activation of FOXC1.

DNA damage, such as double-stranded breaks, can cause genomic instability and has been shown to be an important factor in tumor development. A recent study demonstrated in BRCA1-deficient mammary luminal progenitors that a replication-associated DNA damage response activates NF-κB and leads to hormone-independent proliferation [28]. In addition, we recently reported that FOXC1 is highly expressed in BLBC tumors of patients with BRCA1 mutations [29]. Therefore, further studies are needed to elucidate novel mechanisms or cellular cues for eliciting NF-κB-FOXC1 signaling. In summary, our findings reveal a novel EGFR signaling axis in BLBC. FOXC1 may serve as a read-out of EGFR-NF-κB activity and as a marker for selecting patients who may benefit from anti-EGFR therapy. Blockade of the EGFR-NF-κB-FOXC1 pathway may provide treatment modalities for BLBC and other cancers.

## Methods

### Cell culture

MDA-MB-468, MDA-MB-231, and BT-20 Human BLBC cell lines were purchased from American Type Culture Collection. Cell culture was performed as previously described (Cui et al., 2006). The 2-kb FOXC1-promoter from transcription start site was cloned into the pGL4-luc vector (Promega, Madison, WI).

### Materials

EGF was purchased from Sigma-Aldrich (St. Louis, MO). The MEK inhibitor U0126, AKT inhibitor IV, IKK inhibitor III (BMS-345541), Bay 11–7082, and NF-κB activation inhibitor II (JSH-23) were purchased from Calbiochem (Gibbstown, NJ). For control experiments, cells were incubated with the vehicle dimethylsulfoxide (DMSO) alone. Human FOXC1 siRNA and p65 siRNA were purchased from Santa Cruz Biotechnology (Santa Cruz, CA). Chromatin Immunoprecipitation (ChIP) Kit was purchased from EMD Millipore (Billerica, MA).

### Immunoblotting analysis

Immunoblotting analysis was performed using whole cell lysates prepared in RIPA buffer (50 mM Tris–HCl, pH 7.4, 150 mM NaCl, 2 mM EDTA, 1% NP-40, 10% glycerol) plus a protease inhibitor cocktail (Sigma, St Louis, MO). Nuclear protein was extracted using NE-PER Nuclear and Cytoplasmic Extraction Reagents from Pierce Biotechnology (Rockford, IL). Antibodies anti-p65 (Cat# sc-8008), p50 (Cat# sc-7178), FOXC1 (Cat# sc-21,394), LaminA/C (Cat# sc-7292), β-actin (Cat# sc-8432), GAPDH (Cat# sc-47,724) are from Santa Cruz Biotechnology (Santa Cruz, CA). Antibodies anti-FOXC1 (Cat# 8758), EGFR (Cat# 4267), phosphorylated-EGFR (Cat# 3777), phospho-NF-κB p65 (Ser536) (Cat# 3033) are from Cell Signaling Technology.

### Real-time quantitative reverse transcription-PCR (QRT-PCR)

Total RNA was extracted using the RNeasy Mini Kit (Qiagen, Valencia, CA) according to the manufacturer’s instructions. Reverse transcription was done using the QuantiTect Reverse Transcription Kit. The qRT-PCR assay was done using an iCycler iQ Real-Time Thermocycler (Bio-Rad Laboratories, Hercules, CA). The following primers were used: FOXC1, Forward 5′-GGCAAAGAATTGATCCGGTA-3′, Reverse 5′-TGGATGGCCATGGTGATGAGC-3′; GAPDH, Forward 5′-GATCGAATTAAACCTTATCGTCGT-3′, Reverse 5′-AGCAGCAGAACTTCCACTCGGT-3′, GAPDH was used as internal control.

### Transfection

MDA-MB-468 cells were transfected with the pGL4-FOXC1 promoter reporter construct and the β-galactosidase expression vector pSV-β-Gal (Promega Madison, WI) using Lipofectamine 2000 (Invitrogen, Carlsbad, CA) according to the instruction manual. β-Galactosidase enzyme activity was detected using the β-Galactosidase enzyme assay system with reporter lysis buffer (Promega, Madison, WI). For co-transfection, 500 ng of Flag-ERK2, HA-Myr-Akt1, or HA-Myr-Akt3 constructs were added along with 100 ng of the 2-kb human FOXC1 promoter reporter construct pGL4-FOXC1. For the small interfering RNA (siRNA) experiment, MDA-MB-468 cells were transfected with 30 nM human FOXC1 and p65 siRNA for 48 h, and then treated with EGF for 24 h.

### Chromatin Immunoprecipitation (ChIP) assay

ChIP assay was performed using the EZ-ChIP™ - Chromatin Immunoprecipitation Kit (EMD Millipore, Billerica, MA) according to the manufacturer’s instructions. Antibodies used for the IP include anti-p65 (Santa Cruz Biotechnology Cat# sc-8008), anti-RNA Polymerase II (EMD Millipore Cat# 05-623B), or normal mouse IgG (EMD Millipore Cat# 12-371B). Primers used to detect NF-κB binding sites 1 and 2 and MMP9 promoter were synthesized by Invitrogen. NF-κB binding sites 1 and 2: Forward, 5′- TCACGCACGCTTCTTCGCAG-3′, Reverse, 5′- GAATCCTTGAACCGCCCTCTA-3′ and MMP9 promoter: Forward, 5′- TAAGACATTTGCCCGAGGTC-3′, Reverse, 5′- CTCCCTGACAGCCTTCTTTG-3′.

### Electrophoretic mobility shift assay (EMSA)

After EGF treatment for 24 h, nuclear protein was extracted using NE-PER Nuclear and Cytoplasmic Extraction Reagents (Pierce Biotechnology). EMSA was performed using the LightShift Chemiluminescent EMSA Kit (Pierce Biotechnology) according to the manufacturer’s instructions. The 3′ end biotin-labeled NF-κB probes were synthesized by Invitrogen. NF-κB binding site 1: Forward, 5′-CCGGGAGGGTCTCTCCTCAAGT-3′, Reverse, 5′-ACTTGAGGAGAGACCCTCCCGG-3′ and NF-κB binding site 2: Forward, 5′- GAGCGGGGGCCCTTCCGTGCGT-3′, Reverse, 5′- ACGCACGGAAGGGCCCCCGCTC-3′.

### Biotinylated oligonucleotide precipitation assay

MDA-MB-468 cells were serum-starved overnight and treated with or without 100 ng/mL EGF for 24 h. Nuclear protein was extracted using NE-PER Nuclear and Cytoplasmic Extraction Reagents (Pierce Biotechnology) and incubated with 3′ end biotin-labeled NF-κB probes that were synthesized by Invitrogen overnight at 4 °C. The next day, streptavidin beads were added to this mixture and incubated at 4 °C for 2 h. Beads were washed and p65 expression was tested by immunoblotting.

### Site-directed mutagenesis

Mutation of the two putative NF-κB binding sites in the FOXC1 promoter was performed using the Quik-Change Mutagenesis Kit (Agilent Technologies, Westlake Village, CA). The mutated FOXC1 promoter reporter construct was used for transfection and luciferase assays. The mutation of two NF-κB binding sites was performed using the following primers: Mut-1: Forward, 5′- TTGGGATTCAGCCTCCGGGACCCTCTCTGGTCA AGTCGCTAAAATGC-3′, Reverse, 5′-GCATTTTAGCGACTTGACCAGAGAGGGT CCCGGAGGCTGAATCCCAA-3′ and Mut-2: Forward, 5′-GCACAACGAGCGGCCCCCC TTGGGTGCGTGTCCCCC-3′, Reverse, 5′- GGGGGACACGCACCCAAGGGGGGCC GCTCGTTGTGC-3′.

### Statistical analysis

All experiments were performed 3 times with samples measured in triplicate. Results are expressed as mean ± standard deviation, unless otherwise stated. GraphPad Prism 6.0 software (GraphPad Software, San Diego, CA) was used for statistical analysis.

## Additional files

Additional file 1: Figure S1. NF-κB transcription factor mediates EGF-induced FOXC1 expression in multiple breast cancer cell lines. a MDA-MB-231 and BT-20 cell lines were transiently co-transfected with the FOXC1 promoter-luc and NF-κB (p65), IκBα S32A/S36A super-repressor (p65 + SR-IκBα), or the vector. Reporter activities were assessed by luciferase assays. *, *P* < 0.05; **, *P* < 0.001. b MDA-MB-468 cells were transiently transfected with the IKKβ or SR-IκBα constructs and immunoblotted for FOXC1 expression. c MDA-MB-231 and BT-20 cell lines were treated with 100 ng/mL EGF for 2 h after preincubation with the NF-κB inhibitor Bay 11–7082 for 1 h. FOXC1 mRNA levels were examined using qRT-PCR. **, *P* < 0.001. d Total protein was extracted from MDA-MB-468, MDA-MB-231 and BT-20 cell lines after no starvation or treatment (Left to right, first three lanes) or after serum-starvation overnight with or without EGF treatment for 24 h. EGFR and phosphorylated EGFR (p-EGFR) levels were examined with immunoblotting. e BT-20 cells were treated with 100 ng/mL EGF for 1 h after preincubation with the Akt inhibitor-IV or U0126 (ERK inhibitor) for 45 min. Total protein was extracted and phospho-p65 (Ser 536) was examined by immunoblotting. (TIFF 131 kb)

Additional file 2: Figure S2. Inhibition of NF-κB p65 affects FOXC1 protein levels. MDA-MB-468 cells were serum-starved overnight and treated with EGF for 24 h after pre-incubation with NF-κB inhibitors, Bay 11–7082 or BMS-345541, for 1 h. FOXC1 protein levels were examined by immunoblotting. (TIFF 56 kb)

## Abbreviations

BLBC: Basal-like breast cancer
ChIP: Chromatin immunoprecipitation
DMSO: Dimethylsulfoxide
EGF: Epidermal growth factor
EGFR: Epidermal growth factor receptor
EMSA: Electrophoretic mobility shift assay
ER: Estrogen receptor
FOXC1: Forkhead box C1
HER2: Human epidermal growth factor receptor 2
IKK: IκB kinase
MEFs: Mouse embryonic fibroblasts
NF-κB: Nuclear factor-κB
PR: Progesterone receptor
siRNA: small interfering RNA
SR-IκBα: IκBα S32A/S36A super-repressor
