# Supplementary figures and images for: Identification of EGF-NF-κB-FOXC1 signaling axis in basal-like breast cancer
Source: Cell Commun Signal. 2017 Jun 19;15:22. doi: 10.1186/s12964-017-0180-3 (PMC5477115; doi:10.1186/s12964-017-0180-3)

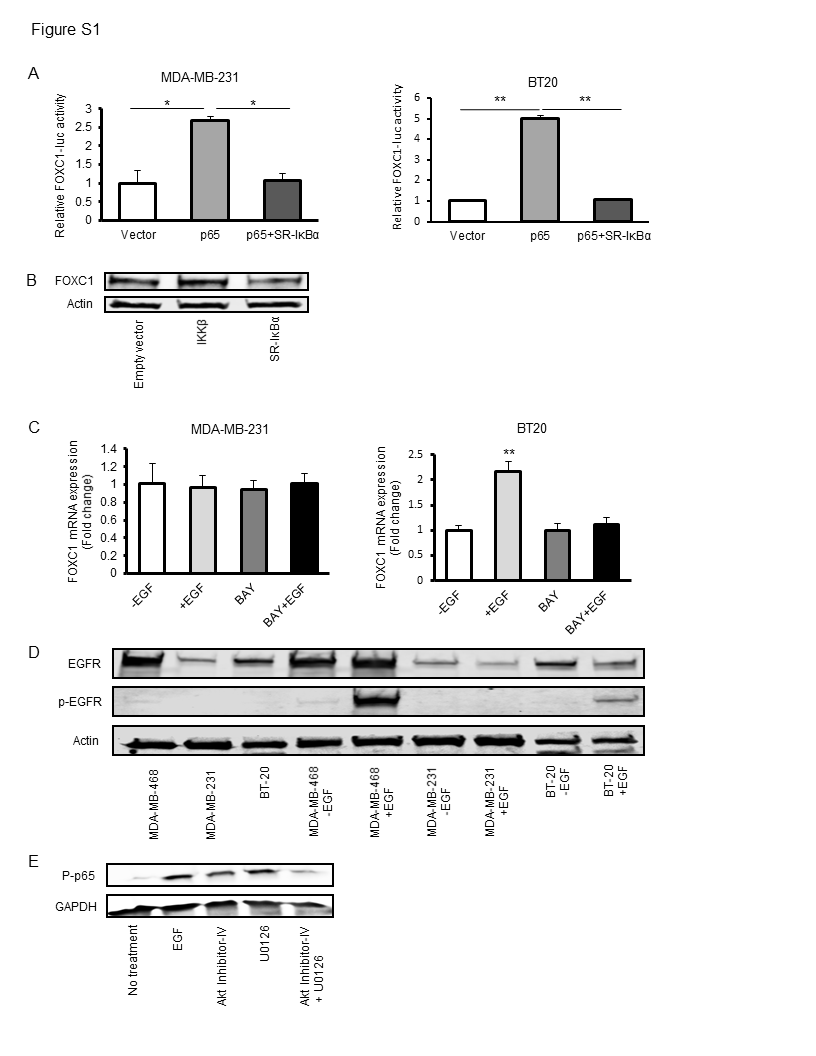

Supplement: Supplementary file 1 — NF-κB transcription factor mediates EGF-induced FOXC1 expression in multiple breast cancer cell lines. a MDA-MB-231 and BT-20 cell lines were transiently co-transfected with the FOXC1 promoter-luc and NF-κB (p65), IκBα S32A/S36A super-repressor (p65 + SR-IκBα), or the vector. Reporter activities were assessed by luciferase assays. *, P < 0.05; **, P < 0.001. b MDA-MB-468 cells were transiently transfected with the IKKβ or SR-IκBα constructs and immunoblotted for FOXC1 expression. c MDA-MB-231 and BT-20 cell lines were treated with 100 ng/mL EGF for 2 h after preincubation with the NF-κB inhibitor Bay 11–7082 for 1 h. FOXC1 mRNA levels were examined using qRT-PCR. **, P < 0.001. d Total protein was extracted from MDA-MB-468, MDA-MB-231 and BT-20 cell lines after no starvation or treatment (Left to right, first three lanes) or after serum-starvation overnight with or without EGF treatment for 24 h. EGFR and phosphorylated EGFR (p-EGFR) levels were examined with immunoblotting. e BT-20 cells were treated with 100 ng/mL EGF for 1 h after preincubation with the Akt inhibitor-IV or U0126 (ERK inhibitor) for 45 min. Total protein was extracted and phospho-p65 (Ser 536) was examined by immunoblotting. (TIFF 131 kb) [file 12964_2017_180_MOESM1_ESM.tif]

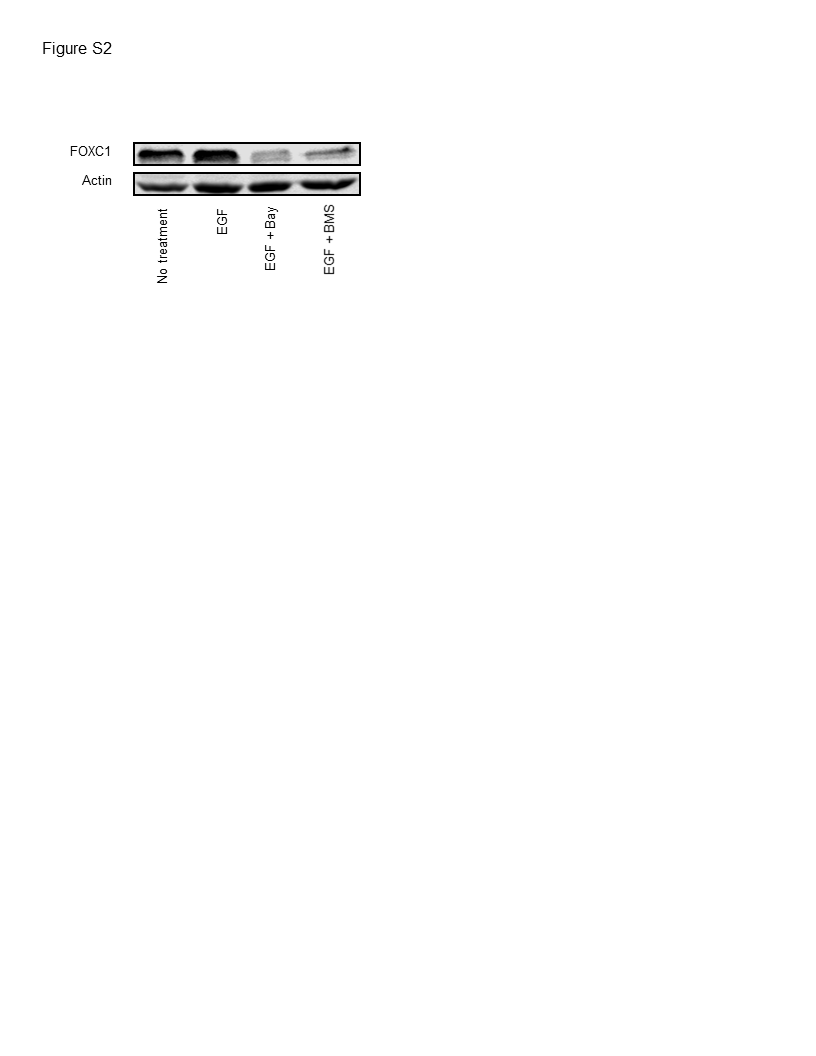

Supplement: Supplementary file 2 — Inhibition of NF-κB p65 affects FOXC1 protein levels. MDA-MB-468 cells were serum-starved overnight and treated with EGF for 24 h after pre-incubation with NF-κB inhibitors, Bay 11–7082 or BMS-345541, for 1 h. FOXC1 protein levels were examined by immunoblotting. (TIFF 56 kb) [file 12964_2017_180_MOESM2_ESM.tif]
